# Supplementary material for: Evidence for Establishing the Clinical Breakpoint of Cefquinome against Haemophilus Parasuis in China
Source: Pathogens. 2021 Jan 22;10(2):105. doi: 10.3390/pathogens10020105 (PMC7912692; doi:10.3390/pathogens10020105)
Supplement: Supplementary file 1 [file pathogens-10-00105-s001.pdf]

Evidence for Establishing the Clinical Breakpoint of Cefquinome against Haemophilus parasuis in China

Kun Mi [1], Da Sun [1], Mei Li [2], Haihong Hao [1,2], Kaixiang Zhou [2], Zhenli Liu [1,2], Zonghui Yuan [1,2,3] and Lingli Huang[1,2,3,*]

[1] National Reference Laboratory of Veterinary Drug Residues (HZAU), Wuhan, 430000, China; mikun@webmail.hzau.edu.cn (K.M.); sundazz@126.com (D.S.); haohaihong@mail.hzau.edu.cn (H.H.); yuan5802@mail.hzau.edu.cn (Z.Y.); liuzhli009@mail.hzau.edu.cn (Z.L.); huanglingli@mail.hzau.edu.cn (L.H.)
[2] MAO Key Laboratory for Detection of Veterinary Drug Residues, Huazhong Agricultural University, Wuhan, 430000, China; hzaulimei@163.com (M.L.); flyingkai@webmail.hzau.edu.cn (K.Z.)
[3] MOA Laboratory for Risk Assessment of Quality and Safety of Livestock and Poultry Products, Huazhong Agricultural University, Wuhan, 430000, China.
* Correspondence: huanglingli@mail.hzau.edu.cn

1. Mice infection experiment

BALB/c mice can eligibly substitute for the piglets to determine the virulence of *H.parasuis* [1]. Mice were challenged by eight strains (serovar 5) of *H.parasuis*, which were chosen from MIC_{90}, with intraperitoneal injection. Due to different infection doses, each strain was divided into 3 groups (n=3) and severally injected 10^7, 10^8, 10^9 CFU/mL with 0.5 mL. The control group (n=3) was challenged by 0.5 mL PBS. The most virulent strain was chosen for PD. The overall score for pathogenicity is based on Yu's study[1]. In brief, different intervals between inoculation and death would be given different scores (i.e., 24 h = 9, 48 h = 6,72 h = 3, and survival = 0), an animal with the most serious gross lesions would be given a total score of 9 (i.e., pleural effusion = 3, peritoneal exudate = 3, and ascites = 3), and an animal with the most serious histopathology would be given a total score of 6 (lung = 3, and spleen = 3).

Anatomical observation of the mice visceral organ characterization and the result can be found in Figure S1. After dissection, a bit of pleural effusion, pulmonary hemorrhage, spleen hemorrhage and swelling in the dead mice compared to the controlling. During 72 h after infection, monitor the number of death mice (Table S1) and observe of the organs after anatomy to evaluate the most virulent strain, *HPS42*, from MIC_{90} selecting for PD experiment.

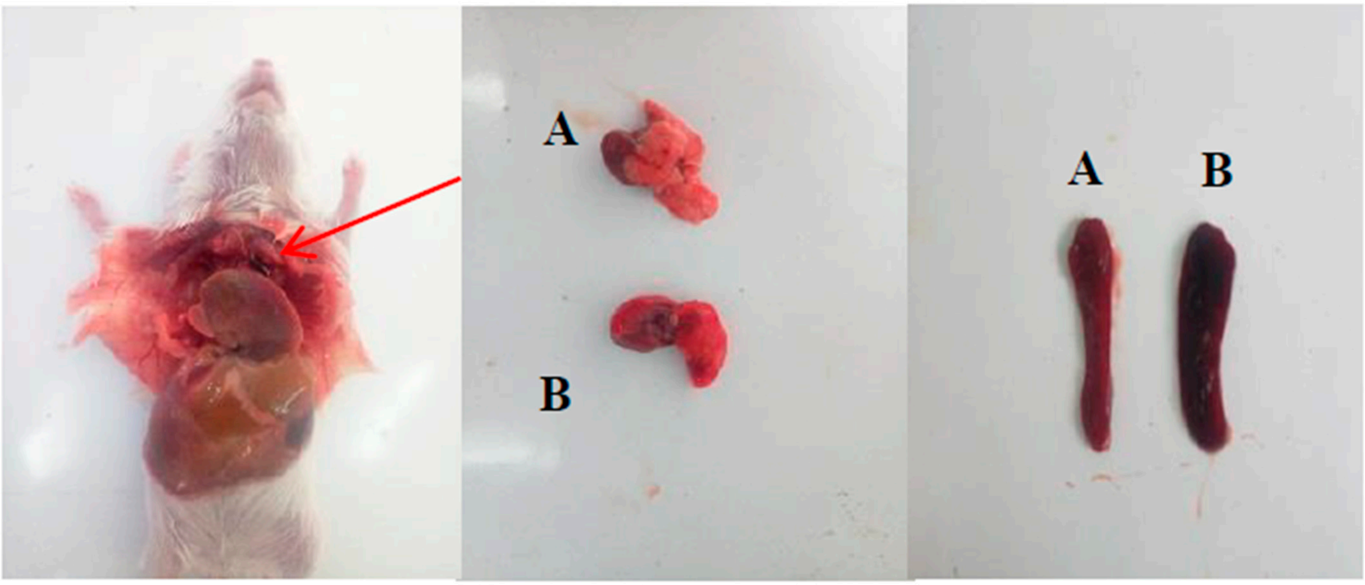

Figure 1. The anatomy of diseased mice (from left right is pleural effusion; lung; spleen).A: Healthy Balb/c mice; B: *H.parasuis* infected Balb/c mice.

Table S1. Virulence results of *H.parasuis* (n=8) for mice by intraperitoneal infection.

Strain	Death			Scored (meaned)		
	1×10^7	1×10^8	1×10^9	1×10^7	1×10^8	1×10^9

	CFU/mL	CFU/mL	CFU/mL	CFU/mL	CFU/mL	CFU/mL
HPS 31	0/3	0/3	3/3	0	2	21
HPS 42	0/3	0/3	3/3	1	2	22
HPS 34	0/3	0/3	3/3	1	1	21
HPS 60	0/3	0/3	1/3	0	1	7
HPS G8	0/3	0/3	1/3	0	0	5
HPS 1006	0/3	0/3	1/3	0	1	6
HPS 35	0/3	0/3	1/3	0	0	5
HPS 12	0/3	0/3	0/3	0	0	0

2. Scores for *H. parasuis* infection in swine

The main observing targets were scored as following criterion described as **Table S2** shown [2]. When the clinical scores reached to the morbidity standards and the pigs exhibited obvious clinical symptoms, such as appetite, willingness to walk, breathing, psychiatric status, and elevated body temperature, the diseased model is successfully established.

Table S2. The scoring system and success criteria for clinical effectiveness study.

Observing target	Scoring systems	Morbidity standard	Cure standard
rectal temperature	0 = less than 39.5°C<br>1 = between 39.5°C–40.5°C<br>2 = higher than 40.5°C	≥ 1	= 0
respiratory state	0 = Rate and pattern normal, no abnormal nasal discharge;<br>1 = Mild-slightly increased breathing rate, some roughness in breathing;<br>2 = Moderate-increased respiratory rate, some abdominal breathing;<br>3 = Severe-increased respiratory rate with abnormal effort-open mouth breathing, regular coughing, grunting	≥ 2	= 0 or ≤ 1
Behavior and depression	0 = Normal-alert, normal appetite, well-hydrated, coat normal;<br>1 = Mild-moves slower than normal, slightly rough coat, may appear lethargic but upon stimulation appears naomal;<br>2 = Moderate-inactive, may be recumbent, standing when gentle stimulated, gaunt, may be dehydrated;<br>3 = Severe-down or reluctant to get up, markedly depressed, gauntness evident, dehydrated	≥ 2	= 0 or ≤ 1

Reference

1. Yu J, Zhang Y, Du Y, Li J, Huang B, Sun W, et al. The BALB/c mouse infection model for improving the Haemophilus parasuis serotyping scheme. *Res. Vet. Sci.* **2016**, *109*, 166–168.
2. Palzer A, Kolb K, Strutzberg-Minder K, Zoels S, Eddicks M, Heinritzi K, et al. Serological course investigations of Haemophilus parasuis and Mycoplasma hyorhinis in three pig farms. Schweizer Archiv fur Tierheilkunde. **2015**, *157*, 97–103.
